# The use of mobile phones for the prevention and control of arboviral diseases: a scoping review

**DOI:** 10.1186/s12889-020-10126-4

**Published:** 2021-01-09

**Authors:** Maria Angelica Carrillo, Axel Kroeger, Rocio Cardenas Sanchez, Sonia Diaz Monsalve, Silvia Runge-Ranzinger

**Affiliations:** 1 Centre for Medicine and Society, Master Programme Global Urban Health, Albert-Ludwigs- University Freiburg, Freiburg im Breisgau, Germany; 2 Institute of Global Health, Heidelberg University, Heidelberg, Germany

**Keywords:** Mobile phone, Mobile technology, mHealth, Dengue, Zika, Chikungunya

## Abstract

**Background:**

The rapid expansion of dengue, Zika and chikungunya with large scale outbreaks are an increasing public health concern in many countries. Additionally, the recent coronavirus pandemic urged the need to get connected for fast information transfer and exchange. As response, health programmes have -among other interventions- incorporated digital tools such as mobile phones for supporting the control and prevention of infectious diseases. However, little is known about the benefits of mobile phone technology in terms of input, process and outcome dimensions. The purpose of this scoping review is to analyse the evidence of the use of mobile phones as an intervention tool regarding the performance, acceptance, usability, feasibility, cost and effectiveness in dengue, Zika and chikungunya control programmes.

**Methods:**

We conducted a scoping review of studies and reports by systematically searching: i) electronic databases (PubMed, PLOS ONE, PLOS Neglected Tropical Disease, LILACS, WHOLIS, ScienceDirect and Google scholar), ii) grey literature, using Google web and iii) documents in the list of references of the selected papers. Selected studies were categorized using a pre-determined data extraction form. Finally, a narrative summary of the evidence related to general characteristics of available mobile health tools and outcomes was produced.

**Results:**

The systematic literature search identified 1289 records, 32 of which met the inclusion criteria and 4 records from the reference lists. A total of 36 studies were included coming from twenty different countries. Five mobile phone services were identified in this review: mobile applications (*n* = 18), short message services (*n*=7), camera phone (*n* = 6), mobile phone tracking data (*n* = 4), and simple mobile communication (*n* = 1). Mobile phones were used for surveillance, prevention, diagnosis, and communication demonstrating good performance, acceptance and usability by users, as well as feasibility of mobile phone under real life conditions and effectiveness in terms of contributing to a reduction of vectors/ disease and improving users-oriented behaviour changes. It can be concluded that there are benefits for using mobile phones in the fight against arboviral diseases as well as other epidemic diseases. Further studies particularly on acceptance, cost and effectiveness at scale are recommended.

**Supplementary Information:**

The online version contains supplementary material available at 10.1186/s12889-020-10126-4.

## Background

Emerging or re-emerging viral diseases such as the most recent coronavirus causing Covid-19 disease or arboviruses transmitted by Aedes mosquitoes causing diseases such as dengue, Zika, chikungunya and yellow fever, represent a significant public health threat in tropical and sub-tropical countries [1]. The rapidly emerging arbovirus infections have reached global scale since the emergence of the chikungunya virus in 2014 and the Zika virus in 2015 in the Americas [2]. Furthermore, dengue continues to be the most prevalent arbovirus disease, with estimates of up to 400 million infections and around 20,000 deaths per year [3, 4]. An indication of this concern was the WHO declaring the increase of microcephaly and Guillain-Barré syndrome caused by Zika, a Public Health Emergency of International Concern [5].

The transmission, epidemiology and clinical symptoms of dengue, Zika and chikungunya are similar, mainly during the acute phase (the first days of the disease [4]); which have produced challenges particularly in the surveillance of these diseases. *Aedes aegypti* (primary vector) and *Aedes albopictus* (secondary vector) transmitting the diseases are widely spread in tropical and subtropical areas [6]. Their high adaptability to urban communities favoured by numerous larval habitats (water containers) [7–10], the abundance of human hosts, climate change and socio-environmental drivers have contributed to the geographical expansion of vector populations [11–13].

The key measure for preventing the transmission continues to be vector control since the first dengue vaccine (CYD-TDV, or Dengvaxia®) is still limited and several others are under development [14]. However, vector control methods have often a limited success rate, not only because of increasing insecticide resistance where chemical methods are used [15, 16], but also because of the lack of community participation and the unsustainability of intensive vector control methods in some countries [17]. The need to prevent the diseases has resulted in new technological innovations including the use of drones with cameras to identify breeding places, computing systems for monitoring and tracking high transmission areas, case tracking of index cases, software for epidemiological surveys and mobile devices for communication and networking [18–20]. These tools may support the management, surveillance, prevention and control of arboviral and other outbreak-prone viral diseases.

One promising tool is the use of mobile phones to support vector control efforts [21]. These devices are widely used and continuously further developed for several health purposes [22–24]. Their use in the health sector is referred to as mobile health (m-health) which is accepted as a component of eHealth. This term has not a single definition, but according to the WHO, eHealth is the use of information and communication technologies for health [25]. Likewise, a standardized definition of mHealth has not been established. However, mHealth, according to the WHO, is defined as a medical and public health practice supported by mobile devices, such as mobile phones, patient monitoring devices, personal digital assistants (PDAs), and other wireless devices [26].

The enormous boom of mobile phones around the world has led to the design of new models with integrated operating systems and other complex functionalities called “smartphones”. Most of these devices are equipped with sensors and modules such as an ambient light sensor, camera, microphone, digital compass, touch-sensitive screen, accelerometer, Bluetooth, Wi-Fi and Global Positioning System (GPS), among others which has promoted various innovative mHealth applications [27, 28]. A variety of applications has aimed at supporting disease surveillance [29], promoting health education and behavioral change [30, 31], supporting the diagnosis [32] as well as improving the treatment and adherence to medication [33], among others. Apart from mobile apps, other mobile phone services (e.g. short message services, SMS) have contributed to improving patient compliance and as appointment reminders [34]. In low- and middle-income countries (LMICs), the use of the mobile technology has been an innovative solution to overcome health barriers such as challenging areas with difficult access, inadequate workforce and restricted financial resources [35, 36].

Despite the potential benefits of mobile phones in health programmes, little is known about their contributions regarding the prevention and control of vector borne diseases, particularly dengue, Zika and chikungunya and no study to date has analysed and summarized the costs or effects in terms of acceptance and effectiveness. Most mobile phone-based studies have focused on other diseases [37–40] and the few studies addressing arbovirus diseases that deployed mobile technology, have not included an analysis of the health outcomes [20, 41].

Therefore, this scoping review has been undertaken to analyse the use of mobile phones as an intervention tool for arbovirus disease programmes focussing on three arboviral diseases transmitted by Aedes mosquitoes: dengue, Zika and chikungunya. We aimed to identify countries where mobile phone-based studies have been conducted, the type of mobile phone services most frequently used, the main purposes of the use of mobile phones as well as to analyse outputs and outcomes regarding performance, feasibility, costs, effectiveness and acceptability.

## Methods

### Scoping review

The scoping review was conducted based on Arksey and O’Malley’s scoping review framework [42]. Additional processual advice, particularly in the identification of relevant studies were used to enhance the selection process of publications [43, 44]. It has been shown that a scoping review is useful to summarize and disseminate research findings and identify research gaps in the existing literature [42, 45]. As opposed to systematic reviews, scoping reviews can include a diversity of sources to map the existing literature in a field of interest in terms of the volume, nature, and characteristics of the primary research [42]. This allows researchers to gain a better overview on a broad topic which has not yet been extensively reviewed or is of complex or heterogeneous nature [46].

The research team consisted of five co-authors with multidisciplinary expertise in infectious diseases, engineering, epidemiology, knowledge of quantitative-qualitative research methods and research synthesis.

### Review question and scope

This scoping review was conducted to answer the question “What is the current evidence of the use of mobile phones as an intervention tool for arboviral disease programmes (namely dengue, Zika and chikungunya) in terms of their acceptance, usability, performance, feasibility, cost and effectiveness?”

Mobile phones are electronic devices used for mobile voice and/or data transmission over a wireless network [47]. A mobile phone is also called a cellular phone or cell phone, but when it is integrated with advanced features similar to a computer it is called a smartphone. This scoping review included mobile phone, smartphone and other mobile phone services such as mobile applications (mobile apps), short message service (SMS), call detail records (CDR) and other mobile phone sensors. Although there are mobile technology-based studies for different health purpose available, there were only a few studies with a high level of evidence, considering output and outcome dimensions. Therefore, this scoping review was particularly interested in providing information on the feasibility of application in the real world, cost, effectiveness and acceptance indicating whether the mobile technology has a realistic chance to be effective, affordable and socially accepted for fighting against arbovirus diseases. Some previous studies that assessed outcome dimensions on technology for health were identified to determine potential outcomes [48–50]. However, modifications and considerations were developed based on included studies.

### Search strategy

The search strategy was conducted through online databases (PubMed, PLOS ONE, PLOS Neglected Tropical Disease, LILACS, WHOLIS, ScienceDirect and Google scholar). Search terms were defined that described two categories: 1) mobile phone technology and 2) arboviral diseases (see Table 1). Each term was separately entered into the advanced search bar from the online database and then combinations were applied following the basic search structure “mobile phone-based terms” AND “arbovirus-based terms” (as appropriate). Medical Subject Headings (MeSH) were used to ensure an accurate search while this option was available in search command. For Google scholar, search the terms “mobile phone” AND dengue Zika chikungunya arbovirus were used to collect a more precise information. A complementary search was performed on Google Web to identify relevant documents in the grey literature (academic reports, theses, and dissertations) which were considered to extend the possible small numbers of published articles in scientific journals. Google search was limited to the 100 most relevant hits. Additionally, the list of references of included articles were used to identify additional sources. Online databases and grey literature were reviewed from 2009 to 2019 in order to assess the most recent evidence of this technological tool. The search was updated on January 12, 2020.

**Table 1:** Search keywords

Category	Keywords
Mobile phone technology	Mobile phone, cellular phone, cell phone, smartphone, mHealth, mobile device, mobile application, SMS, text messaging
Arboviral diseases	Zika, dengue, chikungunya, arbovirus
Time frame	2009–2019

### Inclusion and exclusion criteria

Inclusion criteria were mobile phone studies focusing on 1) dengue, Zika and chikungunya and 2) reporting at least one health outcome relating to costs, effectiveness, acceptability and performance. The review was based on scientific articles using different methodologies (intervention studies, observational studies, pilot studies, qualitative and/or quantitative methods, literature and systematic reviews) as well as grey literature such as academic reports, thesis and dissertations. All articles had to be in English or Spanish published in the last 10 years (January 2009 to December 2019). SMS, text messaging, mobile apps or others mobile service involving mobile phone/smartphone were included. Only studies with a full text were considered.

Exclusion criteria were: 1) study protocols, opinion papers, conference proceedings, reflection articles, letters, book abstracts and posters due to the limitation of the information 2) studies without mention of mobile phone and/or its use, 3) studies without evaluation of the effect of mobile phone interventions 4) Other health areas other than arboviral diseases, 4) Non-English and Non-Spanish language. The inclusion and exclusion criteria are summarized in Table 2.

**Table 2:** Inclusion and exclusion criteria

	Inclusion criteria	Exclusion criteria
Study design	Any randomized or non-randomized study, review articles, meta-analysis, academic reports, thesis and dissertations	Opinion papers, conferences, letters, book abstracts, study protocols
Disease	Dengue, Zika, chikungunya	All other diseases or health conditions
Mobile service delivery	Mobile phone as an intervention tool Short message service (SMS) Mobile applications And other mobile services	The use of mobile phones not specifically analysed
Outcomes	Arboviral disease Acceptance, usability, costs, effectiveness, performance and further findings related to health prevention and control	No outcome assessed
Languages	Spanish, English	All other languages

### Study selection

All retrieved literature was imported into the program Mendeley©; duplicates were identified and deleted. Titles, abstracts and full texts were systematically screened for the inclusion and exclusion criteria during three phases. The first phase of the title screening was conducted to determinate publications which could be discarded when the title was not related to the topic. Publications were included in the second screening round whenever their titles were unclear. In the second phase, all abstracts of the publications that passed the first title screening were read to select relevant information for the purpose of the review publications. Publications without abstracts were included in the third screening round. Finally, the third phase of full text screening was conducted to select publications following the inclusion criteria. By this way only eligible publications in line with the aims of the review were identified for further data extraction. Throughout this whole process, two authors (MAC and AK) made large part of the study selection. A third author (SRR) independently shared work in the screening process. An advisor was consulted in case of doubt whether a publication should be included or not.

### Data extraction

A data extraction form was designed using a Microsoft Excel® spreadsheet. The following information were extracted for each publication: title, author, year, objective, country, target setting, targeted arbovirus disease, study design, mobile phone services (e.g. mobile app, SMS), purpose of the mobile phone for arboviruses (e.g. disease prevention, surveillance), the target users (e.g. health workers), outcome dimension (costs, performance, effectiveness, acceptability). These 12 categories were established based on expert interviews and an initial literature search which was conducted to develop a better classification for achieving the objectives [26, 27, 51]. Given the complexity to classify mobile phone technologies, we iteratively added services if more mobile phone technologies were found that did not fit into any established service (e.g. mobile phone tracking data). We also removed mobile phone services that were not identified in the included studies (e.g. interactive voice response). The data extraction form was optimized based on discussions in our research team. Each full text was reviewed once it was clearly classified with the extraction form. Following the guidelines for conducting a scoping review, no formal assessment of the methodological quality of the included articles was performed [42, 45], however the quality of the papers was defined by the study designs which were eligible for inclusion.

### Synthesis

Themes emerging from the data were analysed and discussed within the research team. Descriptive numerical and thematic analyses are presented as narrative summaries given the heterogeneity of the literature. Narrative summary is a methodology that may involve a simple recounting and description of findings to produce evidence [52]. From the beginning, we were aware that a non-overlapping categorization of individual technologies was difficult due to the complexity of the mobile technology and its integration with other tools. Therefore, publications dealing with more than one mobile phone category were assigned according to the dominant tool; for instance, if a mobile application uses multiple sensors such as Bluetooth or GPS, it is classified as a mobile app, not as a sensor. Last revision was performed to fit each data of the study in their proper category.

## Results

### Results of the study selection process

**Fig. 1:**
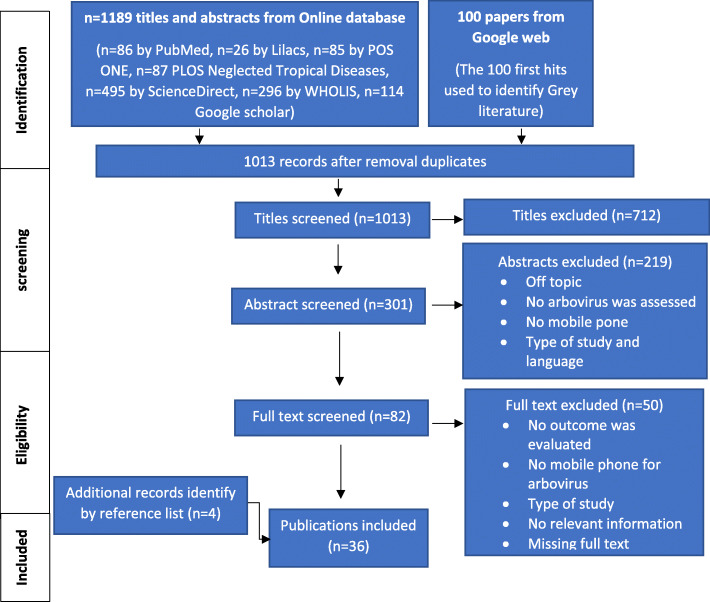
The PRISMA flow diagram. Search results and selection process of studies

A total of 1289 publications were retrieved for this review, including 1189 from the databases and 100 from Google search. After deleting duplicates, 1013 remained for screening the titles, of which 301 were selected for screening of abstracts. After reading abstracts, 82 full texts were considered potentially relevant, of which 32 met our criteria. In addition, 4 papers were identified from reference lists. As a result, 36 studies were included for the data extraction (see Fig. 1). A complete list of all studies can be found in Additional file (see Additional file 1).

### Descriptive results of geographic distribution and study designs

**Fig. 2:**
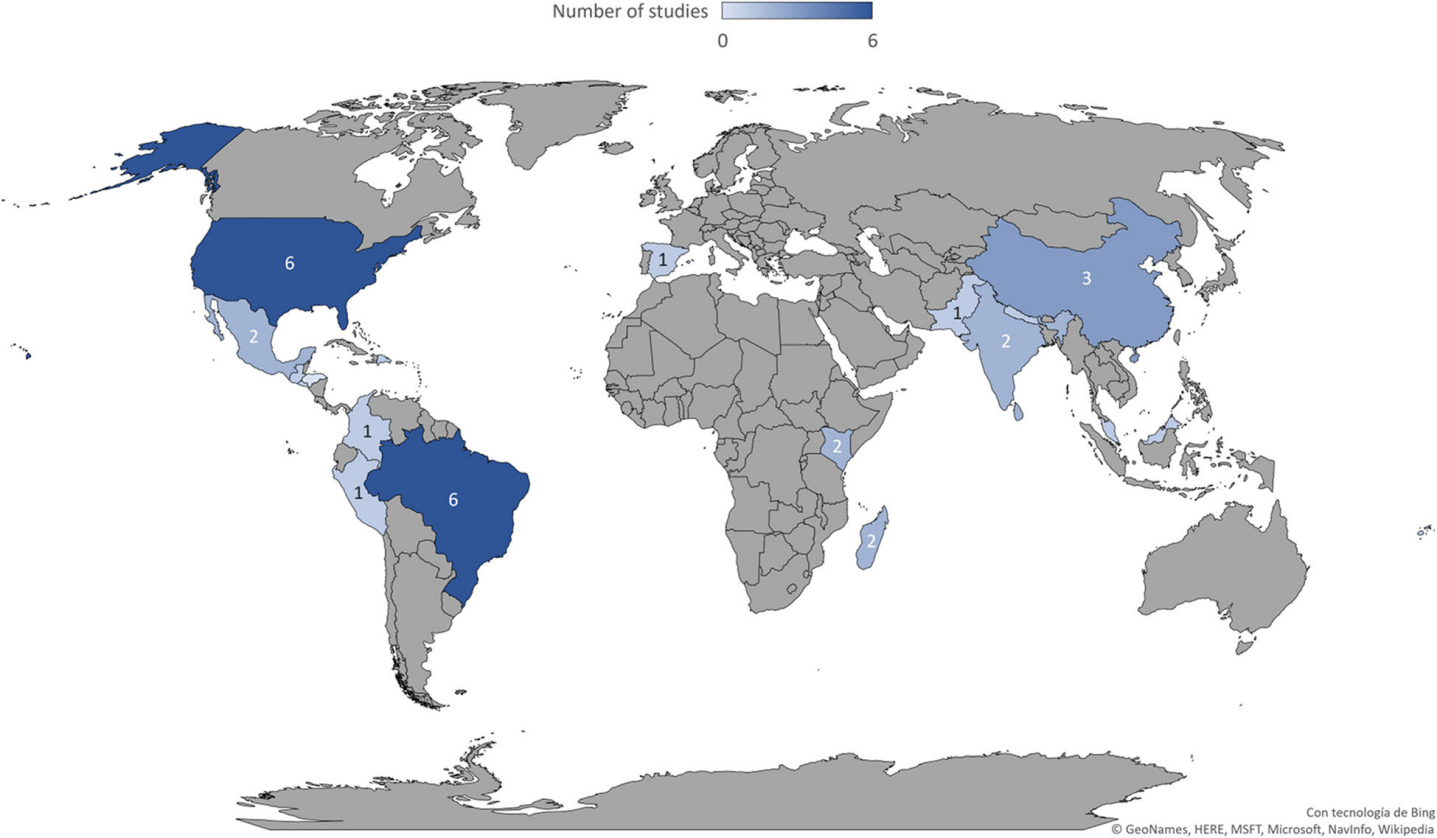
Geographic distribution of mobile phone-based studies. Number of studies per country. This figure shows the distribution of the mobile phone technology used for fighting against arboviral diseases in the last years (from 2009 to 2019). The map was created by our research team using Microsoft® Excel for Microsoft 365 MSO

The included studies came from twenty different countries (see Fig. 2, Geographic distribution of mobile phone-based studies). Eighteen studies were conducted in the American region (Colombia, United States, Brazil, Guatemala, Peru and Mexico), of which one study was conducted in four countries (El Salvador, Honduras, Dominican Republic and Guatemala). Twelve were conducted in the Asia region (Nepal, Singapore, Sri. Lanka, India, China, Malaysia and Pakistan), four in the Africa region (Kenya and Madagascar) and only two were identified in other regions (Fiji and Spain). Most studies were focussing on urban areas where our target diseases are prevalent, only three were specifically conducted in a rural area. Brazil and United States were the countries with the highest number of publications (each one with six), however the studies identified in the United States were not performed under real-world conditions, but rather under controlled conditions, laboratory facilities in particular. Most studies were published in the last three years (*n*=22), reflecting a recent increase in the use of mobile phones for the prevention and control of arbovirus diseases.

From 36 identified studies, most of them had a descriptive approach (*n* = 12), of which some provided preliminary results with small groups of people who “tested” the mobile technology in controlled environments and a few studies described their lessons learned after being conducted at a large scale. Some studies included pilot/feasibility studies (*n* = 6), diagnostic test studies (*n* = 6), retrospective studies (*n* = 4), cross sectional studies (*n* = 4), randomized controlled trials (*n* = 3), quasi-experimental studies (*n* = 2), non-randomized control trial (*n* = 1), and a qualitative study (*n* = 1). Regarding our target diseases, the majority of the 36 studies focused on dengue (*n* = 15), six studies on Zika and no study on chikungunya specifically. However, seven publications covered arboviral diseases in general or *Aedes* vectors. Seven studies on the mobile health technology targeted more than one infectious disease including arboviruses.

### Mobile phone services

With respect to the mobile phone technology, we classified each service of a cell phone to identify which type of mobile phone category were most frequently used in terms of our outcomes. Five mobile phone categories were identified: mobile applications (mobile apps, smartphone apps, mobile software), SMS (Short Message Services), mobile phone tracking data (call detail records, mobile phone signals), camera phone (camera module/image sensor) and simple communication service (calls). An overview is presented in Table 3. The most widely used mobile phone category was mobile applications (*n* = 18). Simple mobile communication (e.g. voice communication) were used less often (see Table 3).

**Table 3:** Mobile phone categories according to the 36 studies

Services	Definition and considerations	Number of hits	References
Mobile applications	Mobile applications, commonly referred to as mobile apps, are software programs designed to run on a mobile device, such as a smartphone or tablet. Many mobile apps have corresponding programs meant to run on desktop computers. This category comprises mobile apps, iPhone apps, smartphone apps, mobile software and m-learning platforms that were run on mobile phone or smartphone.	18	[53–70]
Short message service	Short message service (SMS) is a service for sending electronic message to and from a mobile phone. Messages are usually no longer than 160 alpha-numeric characters and contain no images or graphics. SMS is also known as text messaging.	7	[71–77]
Camera phone	A camera phone is a mobile phone that can take pictures and record video clips. Most new cellular phones are already equipped with cameras which include an image sensor, the lens and microelectronic mechanical system. Smartphone cameras are used for image processing and visual readout.	6	[78–83]
Mobile phone tracking data	Mobile phone tracking data are often call detail records (CDR) that log the location of mobile phone users when they make telecommunication transactions, such as a phone call or text message. This category comprises mobile phone signals.	4	[84–87]
Simple mobile communication	Simple mobile phone communication involves the use of mobile phone numbers to allow contact with others including voice communication (e.g. calls).	1	[88]

### Purpose of mobile phone use in health programmes

To analyse the support that mobile phones are promoting, we noticed that the included studies in this review were focused on four major purposes: surveillance, prevention, diagnosis, and management which are summarized in Table 4. Three studies were identified for both purposes: surveillance and prevention [55, 59, 66], thus those were assigned for both purposes (see Table 4), resulting in 39 studies. This review also identified specific aims in each purpose which are presented in Table 4. Some mobile applications were able to perform more than one aim in surveillance such as data collection, taking mosquitoes photos, geolocation, among others (e.g. The App, Mosquito Alert) [58].

**Table 4:** Mobile phone-based studies by purpose and mobile technology category

Purpose	Specific aims in mobile phones	Mobile phone Service	Application or system’ names/Mobile phone projects	References
surveillance (*n* = 25 studies)	Data collection and reporting cases/ mosquitoes /symptoms to tracking and monitor disease and outbreaks Geolocation of users or breeding sites to identify hotspots Estimation of human movements to predict outbreaks or possible risk areas Capturing vector’ photos, images or sounds to identify mosquito species	mobile apps (*n*=15)	VECTOS system; ^a^OlympTRIP app; Google maps®app; Vigilant-e app; Mosquito Alert; ^a^Mo-Buzz; ^c^MI-Dengue system, Healthy cup app; Abuzz project; Mobile device with OruxMaps, AutoNavi navigation and Baidu Map; ^a^Monitoring app in Fiji; ^b^Chaak system	[53, 55–66, 69, 70]
		SMS (*n*=5)	SMS survey in four countries; SMS for IDSR system in Madagascar; ^b^mSOS project; SMS for sentinel surveillance	[71–75]
		Mobile phone tracking data (*n*=4)	Two studies using CDR in Singapore; mobile phone signals (SS7) in China; CDR in Pakistan	[84–87]
		camera phone (*n*=1)	Smartphone imaged LAMP-OSD assay	[82]
Prevention (*n* = 7)	Health education Promotion of behaviour change in population	mobile apps (*n*=5)	m-learning platform, ^a^OlympTRIP; ^a^Mo-buzz; ^a^Monitoring app in Fiji Mobile social app in India	[54, 55, 59, 66, 67]
		SMS (*n*=2)	SMS conducted in Nepal; SMS conducted in Perú	[76, 77]
Diagnosis (*n* = 6)	Point-of-care diagnosis for detecting viruses of dengue, Zika and chikungunya	camera phone (*n*=5)	Four diagnostic studies using smartphone camera in USA and one in China	[78–81, 83]
		mobile app (*n*=1)	Mobile app for image processing in Malaysia	[68]
Management (*n* = 1)	Communication between health staff and patients for timely diagnosis	simple mobile communication (*n* = 1)	Contact using mobile phone number of patients in India	[88]

^a^Mobile phone projects addressing both surveillance and prevention; ^b^The same mobile phone tool used in two studies; ^c^ The same mobile phone tool used in three studies

In total, the mobile phone-based studies included 25 for surveillance, 7 for disease prevention, 6 for diagnosis and 1 for management (communication). The mobile phone technology, mainly taking advantage of mobile applications, has been most frequently used for multiple aims in surveillance, followed by prevention and diagnosis. The use of mobile phone numbers focussing on communication between health staff and patients was less explored. Short message services were used for surveillance (data collection and reporting) as well as disease prevention (health education and promoting behavioural change). Camera phones coupled to diagnostic platforms and/or assays were aimed at diagnosis of arboviruses and identification of mosquito species.

Among the included studies, we assessed the different target groups or users of the mobile phone technology. Health workers were the main target group for receiving mobile phone services (*n* = 12). This group consisted of vector control staff, healthcare workers, physicians, practitioners, health managers and other health specialists. The second most frequent group were researchers (*n*=11) who conducted studies that used mobile phone tracking data or designed platforms with smartphone cameras under controlled settings. The third most frequent group was the general public (*n* = 9), which includes communities and specific population groups (students, athletes, police officers). Three mobile phone interventions targeted both groups, general public and health workers. Only one mobile phone service was designed for patients.

### Outcome dimensions

This review assessed the following outcome dimensions: performance, acceptance, feasibility, usability, costs and effectiveness. A description is given in Table 5 summarizing the scope of expected outcomes in the 36 studies (see Table 5). Although, the description was developed following prior definitions [48, 49]; some adjustments were developed deductively from the included articles.

**Table 5:** Description of outcome dimensions in 36 studies

Outcome	Description
Performance	Operational characteristics of the mobile phone technology in terms accuracy, completeness, quality data, timeliness, speed, and concordance with other medical reports
Feasibility	The extent to which the mobile health intervention implemented under real conditions can be successfully used in a specific context
Acceptance	User’ attitudes towards the mobile phone technology perceived to be satisfactory and user-friendly.
Usability	Users who are testing the mobile phone technology. This comprises users who downloaded the application/service and used it or active users
Cost	Monetary effort of the use of a mobile technology in a specific context
Effectiveness	Positive effects of mobile phone implementation on public health or health-related behaviour changes.

The analysis of outcome dimensions (Table 6) showed that a large number of studies assessed the performance of their mobile phone services (52%), particularly mobile applications, followed by studies that assessed feasibility (30%). It can be seen that few studies have provided information on acceptance, usability, and effectiveness. Costs analysis or at least estimated prices by mobile phone services were the least explored in this review. Mobile applications were the only service that assessed all outcome dimensions. Usability was only described by mobile apps-based studies. Table 6 summarizes the number of mobile phone services dealing with one or more outcome measurements (see Table 6).

**Table 6:** Number of studies by mobile phone category and outcome dimensions

Mobile phone services	Performance	Feasibility	Acceptance	Usability	Costs	Effectiveness
Mobile applications	9	6	3	5	2	2
Short message service (SMS)	2	2	2	n.a.	1	3
Mobile phone tracking data	2	3	n.a.	n.a.	n.a.	n.a.
Camera phone	5	n.a.	n.a.	n.a.	1	n.a.
Simple mobile communication	1	n.a.	n.a.	n.a.	n.a.	n.a.
	19 (52%)	11 (30%)	5 (13%)	5 (13%)	4 (11%)	5 (13%)

*n.a* not available (No study provided information on that outcome)

### Performance

A variety of operational characteristics were assessed in performance studies. Mobile applications and simple voice communications (calls) reported improvements in terms of completeness, for example, reporting more houses where vector control activities were conducted [53, 56, 88]. Familiarity of health workers with the application and using well known apps (Google maps) and geographic information systems (GIS) helped in locating more houses in real-time. It was also demonstrated that mobile applications were more useful in ensuring data quality and timeliness rather than traditional capturing methods. For instance, Chaak app reported a 19% reduction in the time spent per survey, along with fewer errors in data transfer in comparison with the pen-and-paper data capturing methods [69]. The use of different modes of data transmission from mobile phones to the central server (transference with or without internet), good storing capacity of mobile phones, design of the app (white background and black lettering for better visibility), easy navigation (use of predefined terms, radio buttons and buttons in data entry fields instead of free text input) and trained health workers favoured the good performance of this mobile phone service [70]. Mobile apps also showed good agreement (concordance) between syndromic data reported by participants and by nurses during home visits [57]. Question algorithms with simple terminology and visual aids were key elements to facilitate the self-reporting. The use of smartphones has also led to the development of innovations to identify mosquito species using the acoustic sensor of mobile phones. For example, the Abuzz application was capable of sensitively identifying mosquito species at 10 to 50 mm distance, including *Aedes aegypti* [64].

SMS also demonstrated good performance in terms of completeness. Two studies conducted in Madagascar achieved to transmit more than 70% of patient’s data within 24 h [72, 75]. However, timeliness and data quality were yet an issue depending on the surveillance procedure and capacities of health workers to use SMS. Lack of guidelines and trainings, high workload, and technical problems (e.g. poor telecommunication network) were the main challenges reported by health staff [72].

Mobile phones have been used for tracking users through mobile phone data based on the Signalling System 7 (SS7) and Call Records Details in combination with different datasets (e.g. epidemiological data, environmental data). This mobile phone service showed a strong performance in terms of predictive values, identifying areas with high transmission risk of dengue [86]. Its use has also allowed the integration of mobility models to predict the spread of disease epidemics [85].

Recently, smartphone camera-based diagnostic platforms have been explored to acquire images or read assays such as ELISA tests [68], RT-LAMP reactions [78, 80, 81], RT-PCR and RT-RPA tests [83]. They have demonstrated high accuracy in terms of sensitivity and specificity (range between 95 to 100%) as well as a rapid detection of arbovirus (range between 10 to 20 min) [78–81, 83]. Using a mobile application is an enabler for processing data and interpreting various tests in these diagnostic platforms. For example, Thiha and Ibrahim (2015), developed an ELISA reader for point-of-care dengue detection using the smartphone camera and mobile app. As a result, high performance was demonstrated, with 95% sensitivity and 100% specificity for dengue detection in comparison with standard ELISA microplate readers [68]. However, these prototypes of smartphone-based diagnostic platforms could require qualified personnel to take biological samples and further studies to validate its performance and impact in a real working environment (patient’s home or clinic).

### Feasibility

Mobile apps interventions have been shown to achieve their aims under real conditions. They were particularly used for collecting and transferring entomological information to assess the transmission risk of arboviral diseases. For example, the entomological data (collected by Vectos app, OruxMaps, AutoNavi Navigation and Baidu Map) were analysed in a web platform or central server that successfully identified the level of vector infestation (larval indices) as well as the most abundant breeding sites [53, 65]. Moreover, mobile phones together with traditional methods (ovitraps) and GIS technologies were able to track and monitor mosquitoes, identifying the index of female *Aedes aegypti* [60].

Mobile applications have also proved to be feasible for early detection of arboviral disease based on participatory surveillance which engages users directly in reporting and monitoring of symptoms [55, 57, 63]. This approach required medical staff and scientific experts to validate data reported by users and checked their health status during the intervention. Mobile applications were accompanied by a web-based application or desktop software to facilitate the data management in real time.

Mobile phone tracking data through CDRs and mobile phone signals have also demonstrated to be a feasible service for dengue surveillance in Asia region [84, 86, 87]. This service -when integrated with multiple datasets- has the potential to estimate human mobility in order to predict the spread of arbovirus diseases and outbreaks. Many mobile phones numbers are required to have a better representation of population.

### Acceptance

Mobile apps were generally well received in studies conducted in India, Fiji and Guatemala [57, 66, 67]. General public were the main target group who assessed the acceptance of this mobile phone service. User’s satisfaction with mobile interventions offered was commonly based on how they felt using the app, whether they found it helpful or useful, and whether they would recommend it to others. Although high user satisfaction was reported in most mobile applications, its results depended more on connectivity to the internet and availability of mobile phone in households. For example, a study in Fiji showed positive feedback on user satisfaction in areas with good internet connectivity [66]. Moreover, low socio-economic level of population might be related to people who did not accept the mobile phone intervention [57].

SMS interventions were highly acceptable for the prevention and surveillance of arbovirus. Their acceptability was assessed on how much participants enjoyed the service and whether they perceived it an informative and trustworthy strategy [76]. Another study also checked if their health workers could easily use the service [74]. As result, SMS showed to be a user-friendly service. The participation of stakeholders was useful to promote SMS as media for the prevention of dengue and facilitate its acceptance amongst the community.

### Usability

Most mobile applications showed a good proportion of active users out of all participants who downloaded the app [55, 63, 67], but some researchers recommended more incentives, educational campaigns/trainings and constant communication with study/health personnel to keep to users motivated [57, 59, 63]. Some concerns related to additional expenses of mobile technology (e.g. mobile data plan), mobile phone features (less storage space, slow internet connection), lack of interest and knowledge regarding purpose of mobile phone intervention were associated with a proportion of users who did not use it [67]. Fear and mistrust of adopting a new technology were other reasons for low usability in health workers [59]. In addition, external factors such as period of high staff turnover, cellular tower collapse and socio-politic events caused the decreased use of mobile apps [57].

### Costs

Cost calculations were done in different ways. One study described the market cost of a mobile device [82], another presented estimations of the mobile phone network including calculations of staff salary [70], another estimated the costs of coverage of mobile service during its implementation [58] and another analysed cost-effectiveness for the whole intervention, identifying cost savings [61].

Most studies on costs compared their mobile phone intervention with standards methods for vector surveillance. For example, Mosquito Alert app based on citizen-science initiatives demonstrated a reduction in the cost of coverage in comparation with ovitraps (Mosquito alert costed 1.23 Euros per km2 per month while ovitraps costed about 9.36 Euros per km2 per month). Vector surveillance with ovitraps required much effort to be installed and checked by qualified staff, while mobile application was mostly associated with community buildings and non-recurring investments in technology [58]. Similar economic benefits were briefly mentioned by Bhadra et al. (2018) [82]. However, Chaak app, reported costs equal or slightly higher than traditional capturing methods [70]. Their costs were an issue associated with the type mobile phone network (cost per household were U.S.$0.10 for the pen-and-paper method compared with a cheap mobile phone plan U.S.$0.10 or an expensive mobile phone plan U.S.$2.13 for Chaak app). Additionally, a software developer or a person with technological skills could be required to manage the central server, adding costs to the mobile phone intervention. On the other hand, one study analysed cost-effectiveness of the MI-Dengue system using multivariate models to estimate the median cost savings per case prevented which was median $58 [61]. This system based on the concept that vector control strategies should be applied in targeted areas with higher densities of gravid female mosquitoes, showed a better allocation of resources, saving hundreds of thousands of dollars in direct costs (health care and vector control) as well as lost wages [61]. The cost analysis of this system not only included estimations on mobile phone technology but also costs associated with vector control inspections and other technologies (e.g. computers).

For the diagnosis of arboviral diseases, Chan et al., (2016) mentioned that smartphones are a more affordable alternative to collect fluorescent signals for point-of-care detection of arboviruses in comparison with other portable devices (ESEQuant Tubescanner) [83]. However, information regarding the cost of these diagnostic platforms for point-of-care detection was limited.

### Effectiveness

Few studies showed effective m-health interventions in terms of reducing the vector densities through improved dengue prevention and behaviour change and/or performing as an early warning indicator for outbreaks. The analysis of effectiveness was based on well-defined methodologies (randomized controlled trials or quasi-experimental designs); however, some studies were conducted in specific setting with a short interventional period.

SMS-based studies were the only ones that reported effectiveness in term of improving knowledge and practices of arboviral disease. Preventive messages via mobile phone were able to produce positive changes in human behaviour improving dengue practices and consequently affecting vector densities in households. Dammert et al. (2014), showed that households exposed to repeated preventative messages in Peru reported an increase in the use of vector control methods (window screening and/or mosquito bed nets), and a reduction in the infestation level (e.g. vector water containers testing positive for dengue larvae was 1.44% in the exposed group with SMS vs 2.47% in non-exposed group) [77]. Additionally, SMS with conventional education methods were able to bring a major effect in the prevention of arboviral diseases. In Nepal, SMS together with a prevention leaflet were sent to the community, which increased knowledge and practices of people towards dengue prevention [76]. Availability of mobile phones in households and shared responsibility of the community and other companies were identified as enablers of SMS interventions. In contrast, limited network access in remote areas, reaching private network users and lack of knowledge concerning the purpose of using mobile phones were the main obstacles perceived in the implementation of this mobile phone service.

For surveillance, the use of SMS has also demonstrated to be effective for reporting immediately notifiable diseases [73]. Likewise, mobile applications plus traps were effective for monitoring of *Aedes aegypti* in real time [58, 60, 61]. Their integration with geographic information systems (GIS) enabled the development of early warning mechanisms. For example, GIS datasets obtained from mobile application provided early warning signals in low endemicity areas where traditional surveillance was limited [58]. Positive results were also observed in MI-Dengue system using a website platform, a mobile device (plus mosquito traps) and vector control inspections. Researchers showed that, in Brazil, the system was able to identify high risk areas which were then targeted for vector control and consequently prevented 27,191 cases of dengue fever [61]. Using both approaches together (standard surveillance methods and mobile apps) are effective as entomological surveillance instruments for decision-making in the control of Aedes mosquitoes and subsequent action.

## Discussion

### Overview of findings

This scoping review presents evidence on mobile phone technology regarding dengue, Zika and Chikunguny*a. mobile* applications, short message services, phone camera, mobile phone tracking data and simple mobile communication are mostly being used in urban areas of American and Asian countries, which is consistent with the high burden of three arboviruses reported in these regions [89]. Our review shows that the most dominant purpose is surveillance while research on management of arboviral disease is limited (see Table 4) which is consistent with another review [90]. However, we also observed an increasing interest in the use of mobile phones addressed to diagnose arboviral diseases in the last years. Mobile application is the most popular mobile phone service for combating arboviral diseases. Given the capacities of smartphones, health programmes have taken advantage of mobile apps to respond to different needs [91].

### Benefits by purpose of mobile phones

The current evidence shows benefits of mobile phone services in the fight against arboviruses that go beyond improving user-provider communication. For surveillance, mobile applications and SMS showed to be useful in data collection, including reduction in error of transcribed data, rapid data transfer to a central server, and good completeness in terms of more dengue case reporting. Mobile applications, plus a web application and GIS technologies were enabler to monitor disease/mosquitoes and geolocate hotspots, facilitating the analysis of entomological indices and the production of case distribution maps. Evidence also identified that mobile apps with other approaches (participatory surveillance) and traditional vector control methods (traps for mosquitoes) demonstrated to be an effective complementary tool as an early warning indicator for arboviral disease outbreaks [58, 60] and thus triggering an early outbreak response [61]. This finding substantiates the results of earlier conclusions, where the combination with other approaches helped improve health outcomes [92]. Likewise, reliable self-reporting of symptoms and high ability to identify mosquitoes were other benefits in mobile apps. On the other hand, mobile phone tracking data in combination with other datasets demonstrated good accuracy to predict areas at risks and outbreaks which is evidenced in previous studies [93, 94]. A well-organized coordination of local governments and telecommunication companies is important for enhancing data utilization [95].

For diagnosis, camera phone/smartphone in conjunction with a medical test demonstrated an adequate resolution of images and rapid readout of assays. Although, the use of smartphones for diagnosis is a rapidly evolving area [96]. This technology has so far only been tested under controlled conditions. Further validation and analysis are needed to understand by whom and where these may be used.

For the disease prevention, SMS has shown to be effective for promoting behavioural changes in the community, with evidence of decrease vector densities [77]. This mobile phone service in combination with other conventional media increase the knowledge and preventive practices in the population [76]. This finding is similar with a study conducted in the United Kingdom that reported positive results using SMS and other promotional media [97]. However, it is needed to select media that suits the target group.

### Findings of outcome dimensions

In general, mobile phone interventions showed good performance for different aims. We observed that some features of mobile phones such as interface design, navigation and terminology were an enabler to boost operational characteristics of mobile phones (e.g. data quality), which support other studies [98–100]. In addition, other building capacities such as familiarity of users to use the mobile phone service, good wireless data service, participation of stakeholders, coordination with multiple sectors and integration with other existing technologies, were key to ensure good performance of mHealth intervention and improve other outcome dimensions (feasibility and effectiveness). We identified some barriers such as weak internet connectivity, low phone ownership and poor cell phone network access which still represent a challenge for some portions of the population [101–103]. Cultural and socio-demographic factors (age, gender, education, among others) can also influence the adoption of a mobile technology [104, 105]. Thereby, this technology should be implemented according to local realities and needs of the community [106].

Good usability and acceptance were reported in most mobile apps and SMS interventions, but some efforts were required to keep users motivated. Aitken & Lyle (2017) suggests offering incentives based on use and user compliance to improve health outcomes [107], but the provision of mobile phone is probably an unnecessary strategy to improve usability [57, 74]. This review identified other barriers such as mistrust of users, lack of trainings and technological problems that affected the usability and other outcomes dimensions which are commonly reported in other studies [108, 109].

Although, few information was found on costs of mobile phone technology in arboviruses. The evidence shows that costs may depend on the mobile phone network and the initial investment of the intervention. According to WHO, mHealth focused on disease surveillance needs to invest in technological capabilities (e.g. computers, software developers) [26]. However, mHealth programmes have reported to be sustainable in the management of other diseases [110]. Further outcome dimensions need to be evaluated that go beyond the performance of the mobile phone in order to understand their real impact on arboviruses.

### Recommendations

The following recommendations can be given for implementing mHealth interventions for combating arbovirus and other infectious diseases (see Table 7):

**Table 7:** Recommendations for implementing a mobile phone intervention

*Key recommendations* Applications should be developed with friendly designs, easy navigability, simple language, and visual aids to help enhance the operational characteristics of mobile phone service. According to the purpose and complexity of mHealth intervention, qualified staff with technological skills will be required to manage the central server and other technological needs (e.g. software developers). Coordination with telecommunication companies will be an enabler to reach private network users and improve data utilization. Initial information on mobile phone penetration, connectivity of the internet, environmental, socio-demographic conditions, and other external factors (e.g. staff turnover) should be analysed before starting an mHealth programme. Active participation of stakeholders facilitates the acceptance and promotion of mobile strategy amongst target groups. Use of existing technologies (e.g. Google Maps and GIS) could be helpful and flexible for tracking and geolocation of arboviruses. Regular training and supervision are required to increase capacities and confident of health staff to use the mobile phone. Promoting the download of mobile applications onto the mobile phones of users can potentially reduce costs and be sustainable for participatory surveillance programmes. Constant communication between general public and health staff can be enhanced by the use of mobile applications for participatory surveillance. Encourage users to participate in mobile phone intervention by providing some small incentive and ensuring that the download and use is for free. Use of traditional vector surveillance methods (ovitraps or adult traps) combined with the mobile phone technology can produce better results for early warming information for hotspots of disease transmission. Investing more promotional and educational efforts could be required to increase usability, improve social engagement and raise awareness The combination of the mobile phone technology with promotional media can provide better results for increasing knowledge and affecting positively behaviour in people toward prevention of arboviral diseases.	

### Limitations

Although the scoping review was conducted in line with the guidelines of the methodology, we still need to acknowledge some limitations. Some articles might not have been identified due to the language restriction (English and Spanish), or indexed with English key terms. This may have resulted in an underrepresentation of some geographic regions, such as French speaking Africa. The importance of these exclusions is unknown.

The majority of scoping reviews did not assess the methodological quality of the individual studies [111, 112]. However, looking at all 36 studies included in this review, it was noticeable that some mobile technologies were conducted in a short period of time and tested with small sample size. This made it difficult to assess their relevant information. Due to the shortage of randomized trials, this review could not provide much information about the effectiveness of the mobile technology in terms of contributing to reduce vector density and disease incidence. Further studies over an extended period of time and in diverse settings is necessary to understand the long-term influence of the intervention implemented in this study. The diversity of mobile phone programmes including different approaches and procedures made it difficult to compare and identify the most effective, acceptable and affordable mobile phone category. However, we described characteristics, purposes and benefits of using mobile phones in arboviral disease programmes which can be adapted to specific user needs.

## Conclusions

This scoping review describes how mobile phones are leveraged in the fight against arboviral diseases. Thirty-six publications coming from twenty countries were described using different mobile phone services: mobile applications, short message services (SMS), phone camera, mobile phone tracking data and simple mobile communication. In the last decade, mobile phone technology has been used to enhance surveillance, prevention, diagnosis, and arbovirus management. Most interventions that involved a mobile phone reported positive results in terms of outcome dimensions (performance, feasibility, acceptance, usability, costs and effectiveness). Further studies at a larger scale are required to assess the impact with more precision.

## Supplementary Information

**Additional file 1.**

## Data Availability

The datasets used and/or analysed during the current study are available from the corresponding author on reasonable request.

## Abbreviations

App: Application
CDRs: Call detail records
ELISA: Enzyme-linked immunosorbent assay
GPS: Global positioning system
GIS: Geographic information system
mHealth: Mobile health
M-learning: Mobile learning.
RT-LAMP: Reverse transcription-loop-mediated isothermal amplification
RT-PCR: Reverse transcription polymerase chain reaction
RPA: Recombinase polymerase amplification
SMS: Short message service
WHO: World health Organization
