# Supplementary material for: The use of mobile phones for the prevention and control of arboviral diseases: a scoping review
Source: BMC Public Health. 2021 Jan 9;21:110. doi: 10.1186/s12889-020-10126-4 (PMC7796697; doi:10.1186/s12889-020-10126-4)
Supplement: Supplementary file 1 — Additional file 1. [file 12889_2020_10126_MOESM1_ESM.docx]

**Overview of all included studies**

| # | Title | Author(s) | Year | Country | Description |
| --- | --- | --- | --- | --- | --- |
| 1 | The addition of mobile SMS effectively improves dengue prevention practices in the community: an implementation study in Nepal | Bhattarai AH, Sanjaya GY, Khadka A, Kumar R, Ahmad RA | 2019 | Nepal | Communities received SMS and dengue preventative leaflets for 6 weeks to improve knowledge and dengue practices |
| 2 | VECTOS: An Integrated System for Monitoring Risk Factors Associated with Urban Arbovirus Transmission | Ocampo CB, Mina NJ, Echavarria MI, Acuña M, Caballero A, Navarro A, Aguirre A, Criollo IS, Forero F, Azuero O, Alexander ND | 2019 | Colombia | VECTOS is a web-based information system with two mobile apps to capture entomological and social and demographic information of population |
| 3 | Using Human Movement Data to Identify Potential Areas of Zika Transmission: Case Study of the Largest Zika Cluster in Singapore | Rajarethinam J, Ong J, Lim SH, Tay YH, Bounliphone W, Chong CS, Yap G, Ng LC | 2019 | Singapore | An origin–destination model was developed using CDRs from mobile phone subscribers to quantify human movements, thus identify transmission areas |
| 4 | Assessing the interplay between human mobility and mosquito borne diseases in urban environments | Massaro E, Kondor D, Ratti C | 2019 | Singapore | Four mobility models were compared using mobile phone records to estimate people’s movements |
| 5 | Use of short message service for monitoring Zika-related behaviors in four Latin American countries: lessons learned from the field | Kumoji E, Khan Sohail S | 2019 | Multiple countries in Latin-American | SMS surveys were conducted in four countries to capture changing community perceptions of risk and behaviours to prevent Zika |
| 6 | The prevention of arboviral diseases using mobile devices: a preliminary study of the attitudes and behaviour change produced by educational interventions | Abel Mangueira FF, Smania-Marques R, Dutra Fernandes I, Alves Albino V, Olinda R., Acácia Santos-Silva T, Traxler J, Matheson D, Santos S | 2019 | Brazil | Police officers and students participated in a learning platform using mobile devices to assess knowledge, attitudes and behaviours related to the prevention of arboviruses |
| 7 | Direct nucleic acid analysis of mosquitoes for high fidelity species identification and detection of Wolbachia using a cell phone | Bhadra S, Riedel TE, Saldaña MA, Hegde S, Pederson N, Hughes GL, Ellington AD. | 2018 | United States | LAMP-OSD assay achieved to amplify nucleic acids to be visually read and images acquired using a camera cell phone |
| 8 | Mobile based surveillance platform for detecting Zika virus among Spanish Delegates attending the Rio de Janeiro Olympic Games | Rodriguez-Valero N, Luengo Oroz M, Cuadrado Sanchez D, Vladimirov A, Espriu M, Vera I, Sanz S, Gonzalez Moreno JL, Muñoz J, Ledesma Carbayo MJ. | 2018 | Brazil | Athletes and trainers were monitored using OlympTRIP, a participatory surveillance platform that included a mobile app to check their health status |
| 9 | Feasibility of mapping of dengue fever patients admitted to medical wards of Teaching Hospital Karapitiya using Google maps®app in mobile phones or tablet | Hewavithana JS, Palangasinghe DR, Dahanayaka NJ | 2018 | Sri Lanka | Positive dengue patients were recruited using Google maps® app to identify geographical locations |
| 10 | Evaluation of the reinforced integrated disease surveillance and response strategy using short message service data transmission in two southern regions of Madagascar, 2014-15 | Randriamiarana R, Raminosoa G, Vonjitsara N, Randrianasolo R, Rasamoelina H, Razafimandimby H, Rakotonjanabelo AL, Lepec R, Flachet L, Halm A | 2018 | Madagascar | The integrated disease surveillance and response system reinforced used SMS to data transfer by health workers |
| 11 | Timely diagnosis, use of information technology and mosquito control prevents dengue outbreaks: Experience from central India | Barde PV, Mishra N, Singh N | 2018 | India | Vector staff used the contact mobile number of the positive cases to track patients and conduct vector control activities within 24 hours |
| 12 | Simpler, Faster, and Sensitive Zika Virus Assay Using Smartphone Detection of Loop-mediated Isothermal Amplification on Paper Microfluidic Chips | Kaarj K, Akarapipad P, Yoon JY | 2018 | United States | A platform with a paper microfluidic chip was developed to identify Zika and other arboviruses. Visible colour changes were observed and quantified by smartphone imaging |
| 13 | Smartphone-Based Fluorescent Lateral Flow Immunoassay Platform for Highly Sensitive Point-of-Care Detection of Zika Virus Nonstructural Protein 1 | Rong Z, Wang Q, Sun N, Jia X, Wang K, Xiao R, Wang S | 2018 | China | Portable platform was developed for point-of-care detection of Zika. Fluorescent signals were read out by the smartphone camera. |
| 14 | Performance of a Mobile Phone App-Based Participatory Syndromic Surveillance System for Acute Febrile Illness and Acute Gastroenteritis in Rural Guatemala | Olson D, Lamb M, Lopez MR, Colborn K, Paniagua-Avila A, Zacarias A, Zambrano-Perilla R, Rodríguez-Castro SR, Cordon-Rosales C, Asturias EJ | 2017 | Guatemala | Community people used Vigilant-e, a symptom diary app to submit weekly health reports using simplified question algorithms |
| 15 | Citizen science provides a reliable and scalable tool to track disease-carrying mosquitoes | Palmer JRB, Oltra A, Collantes F, Delgado JA, Lucientes J, Delacour S, Bengoa M, Eritja R, Bartumeus F. | 2017 | Spain | Mosquito Alert used an android mobile app to identify tiger mosquitoes by combining citizen scientists' observations (optional photos) with expert validation |
| 16 | Lessons from the Implementation of Mo-Buzz, a Mobile Pandemic Surveillance System for Dengue | Lwin MO, Jayasundar K, Sheldenkar A, Wijayamuni R, Wimalaratne P, Ernst KC, Foo S | 2017 | Sri Lanka | An android mobile-based system was developed with two versions, i) health workers and ii) general public |
| 17 | Intelligent monitoring of Aedes aegypti in a rural area of Rio de Janeiro State, Brazil | Sanavria A, Silva CB, Electo ÉH, Nogueira LC, Thomé SM, Angelo ID, Vita GF, Sanavria TE, Padua ED, Gaiotte DG | 2017 | Brazil | Intelligent Dengue Monitoring System (MID) used to capture and identify mosquitoes in real time. This consists of MosquiTRAPS and a specific software to send field data by cell phones directly to a Web-based database. Here studies assessed vector infestations in 19 locations |
| 18 | Cost-effectiveness of Novel System of Mosquito Surveillance and Control, Brazil | Pepin KM, Marques-Toledo C, Scherer L, Morais MM, Ellis B, Eiras AE | 2013 | Brazil | MID was conducted in 21 cities. Traps for adult female mosquitoes were distributed throughout each city. Some cities were selected to use MID and other as control (only) |
| 19 | Preliminary evaluation of the "Dengue-MI" technology for Aedes aegyptimonitoring and control | Eiras AE, Resende MC | 2009 | Brazil | Traps were set up in houses. Three comparative clusters were formed in the three municipalities (the first with the cities that partially adopted MID, the second with those that fully adopted MID, and the third with those that only used larval surveys) |
| 20 | Saúde na Copa: The World's First Application of Participatory Surveillance for a Mass Gathering at FIFA World Cup 2014, Brazil | Leal Neto O, Dimech GS, Libel M, de Souza WV, Cesse E, Smolinski M, Oliveira W, Albuquerque J | 2017 | Brazil | Healthy cup is a mobile app to report health condition of users. This app contains a list of 10 symptoms for real-time identification of possible infectious diseases, including dengue |
| 21 | Hands-free smartphone-based diagnostics for simultaneous detection of Zika, Chikungunya, and Dengue at point-of-care | Ganguli A, Ornob A, Yu H, Damhorst GL, Chen W., Sun F, Bhuiya A, Cunningham BT, Bashir R | 2017 | United States | Point-of-care platform using a smartphone to acquire real-time images of the amplification reaction and displaying a visual read-out of the essay |
| 22 | A smartphone-based diagnostic platform for rapid detection of Zika, chikungunya, and dengue viruses | Priye A, Bird SW, Light YK, Ball CS, Negrete OA, Meagher RJ | 2017 | United States | A portable “LAMP box” with a smartphone which employed a novel algorithm using mobile app to analyse fluorescent signals |
| 23 | Understanding mSOS: A qualitative study examining the implementation of a text-messaging outbreak alert system in rural Kenya | Toda M, Njeru I, Zurovac D, Kareko D, O-Tipo S, Mwau M, Morita K | 2017 | Kenya | Health workers and managers assessed a mobile SMS-based disease outbreak alert system called mSOS through a qualitative study |
| 24 | Using mobile phones as acoustic sensors for high-throughput mosquito surveillance | Mukundarajan H, Hol FJH, Castillo EA, Newby C, Prakash M | 2017 | United States | Abuzz project is a mobile app that collects acoustic data to identify mosquito species with maximum sensitivity in audio acquisition. |
| 25 | Mosquito (Diptera: Culicidae) Habitat Surveillance by Android Mobile Devices in Guangzhou, China | Wu TP, Tian JH, Xue RD, Fang YL, Zheng AH | 2016 | China | Four areas were selected for vector surveillance using Android mobile devices with a camera, GPS and mobile apps such as OruxMaps, AutoNavi Navigation and Baidu Map |
| 26 | Attitudes and Perceptions of Medical Undergraduates Towards Mobile Learning (M-learning) | Patil RN, Almale BD, Patil M, Gujrathi A, Dhakne-Palwe S, Patil AR, Gosavi S | 2016 | India | Students receive information on several diseases, including dengue through a conventional class and a group social app which provided learning materials |
| 27 | Mapping intra-urban transmission risk of dengue fever with big hourly cellphone data | Mao L, Yin L, Song X, Mei S | 2016 | China | Mobile phone data were generated from cell phone signals based on the the Signaling System 7 (SS7) which is an active tracking strategy in combination with the random forest classification and mosquito activities |
| 28 | Effectiveness of a Mobile Short-Message-Service–Based Disease Outbreak Alert System in Kenya | Toda M, Njeru I, Zurovac D, O-Tipo S, Kareko D, Mwau M, Morita K | 2016 | Kenya | The system mSOS was tested to determine the effectiveness of a SMS-based system used by health workers and managers for the timely notification of disease outbreaks, including dengue. |
| 29 | Rapid, Affordable and Portable Medium-Throughput Molecular Device for Zika Virus | Chan K, Weaver S, Wong P, Lie S, Wang E, Guerbois M, Vayugundla SP, Wong S | 2016 | United States | A smartphone camera was used to record fluorescent signal change over time during the RT-RPA reactions. |
| 30 | Impact of human mobility on the emergence of dengue epidemics in Pakistan | Wesolowski A, Qureshi T, Boni MF, Sundsøy PR, Johansson MA, Rasheed SB, Engø-Monsen K, Buckee CO | 2015 | Pakistan | CDRs were analysed in combination with dengue data and climate information to estimate human mobility |
| 31 | A Colorimetric Enzyme-Linked Immunosorbent Assay (ELISA) Detection Platform for a Point-of-Care Dengue Detection System on a Lab-on-Compact-Disc | Thiha A, Ibrahim F | 2015 | Malaysia | Smartphone application used in the diagnostic platform to display ELISA tests results that were transmitted via Bluetooth. |
| 32 | Mobile Application for Dengue Fever Monitoring and Tracking via GPS: Case Study for Fiji | Reddy E, Kumar S, Rollings N, Chandra R | 2015 | Fiji | A mobile application to monitor Dengue based on a global positioning system (GPS) was designed to check for symptoms and self-report dengue cases. |
| 33 | Preventing dengue through mobile phones: evidence from a field experiment in Peru | Dammert AC, Galdo JC, Galdo V | 2014 | Peru | Families received dengue preventive messages during 3 months before the peak of the dengue season |
| 34 | Cell phone-based system (Chaak) for surveillance of immatures of dengue virus mosquito vectors | Lozano-Fuentes S, Wedyan F, Hernandez-Garcia E, Sadhu D, Ghosh S, Bieman JM, Tep-Chel D, García-Rejón JE, Eisen L | 2013 | Mexico | The Chaak system (mobile phone-based system) was tested by a small group of workers to assess its costs. |
| 35 | Using Cell Phones for Mosquito Vector Surveillance and Control | Lozano–Fuentes S, Ghosh S, Bieman J, Sadhu D, Hernandez–Garcia E, Garcia–Rejon J, Wedyan F, Tep-Chel D, Eisen L | 2012 | Mexico | Mobile phone with android system called Chaak was used by a vector surveyor receiving tasks and capturing the data to assess the performance |
| 36 | Sentinel surveillance system for early outbreak detection in Madagascar | Randrianasolo L, Raoelina Y, Ratsitorahina M, Ravolomanana L, Andriamandimby S, Heraud JM, Rakotomanana F, Ramanjato R, Randrianarivo-Solofoniaina AE, Richard V | 2010 | Madagascar | Practitioners (at least two by health centre) transmit patient data using short message services (SMS) |
